# Molecularly imprinted polymers *via* reversible addition–fragmentation chain-transfer synthesis in sensing and environmental applications†

**DOI:** 10.1039/d2ra00232a

**Published:** 2022-03-23

**Authors:** Irvin Veloz Martínez, Jackeline Iturbe Ek, Ethan C. Ahn, Alan O. Sustaita

**Affiliations:** School of Engineering and Science, Tecnologico de Monterrey Av. Eugenio Garza Sada 2501 Monterrey N.L. 64849 Mexico alan.sustaita@tec.mx; Department of Electrical and Computer Engineering, The University of Texas at San Antonio San Antonio TX 78249 USA

## Abstract

Molecularly imprinted polymers (MIP) have shown their potential as artificial and selective receptors for environmental monitoring. These materials can be tailor-made to achieve a specific binding event with a template through a chosen mechanism. They are capable of emulating the recognition capacity of biological receptors with superior stability and versatility of integration in sensing platforms. Commonly, these polymers are produced by traditional free radical bulk polymerization (FRP) which may not be the most suitable for enhancing the intended properties due to the poor imprinting performance. To improve the imprinting technique and the polymer capabilities, controlled/living radical polymerization (CRP) has been used to overcome the main drawbacks of FRP. Combining CRP techniques such as RAFT (reversible addition–fragmentation chain transfer) with MIP has achieved higher selectivity, sensitivity, and sorption capacity of these polymers when implemented as the transductor element in sensors. The present work focuses on RAFT-MIP design and synthesis strategies to enhance the binding affinities and their implementation in environmental contaminant sensing applications.

## Abstract 2

Environmental contaminants constitute an ecological and health hazard, which requires green sensing. The RAFT-MIP approach for tailor-made selective receptors enhances them *via* binding affinities for use in environmental contaminant sensors.

## Graphical abstract

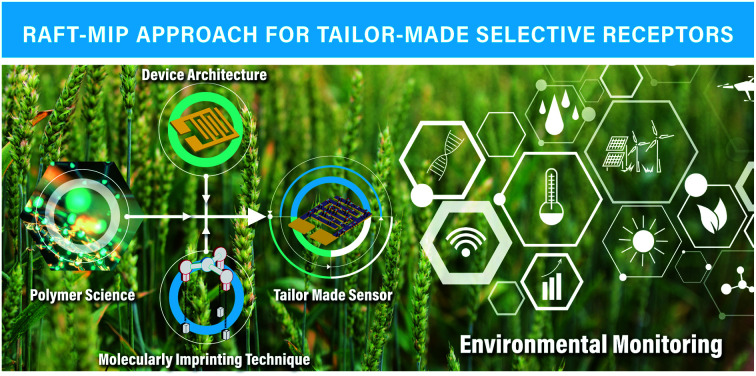

## 1. Introduction

The increasing uncontrolled release of chemicals from primary activities and industrial processes has negatively impacted the environment and human health. For example, the constant exposure to cancer causing or promoting agents such as glyphosate is a common situation in communities near agriculture fields.^1–3^ The affected environmental elements also include water, soil, and atmosphere, implying a broad negative impact on human health.^4^ Analytical instrumentation has been implemented to address this situation by sporadically keeping track of the level of free contaminants in urban systems.^5^ However, this approach requires a long time between sampling and quantification, thus making it irrelevant to the real situation.^6,7^ The environment is dynamic and complex, and our ability to detect and neutralize pollutants must adapt.^8–11^

Numerous analytical methods for quantification, including liquid chromatography (LC), atomic absorption spectroscopy (AAS), and gas chromatography (GC), have been reported for the determination of pollutants in different scenarios from *in situ* analysis stations.^12–15^ However, the fixed costs related to this kind of infrastructure make this solution not financially viable. *In situ* quantification techniques have been developed as an alternative to solve this challenge.^16–18^ The application of the lab-on-a-chip principle is the most promising since its objective is to achieve laboratory grade precision and accuracy with transducing devices. Although these *in situ* detection methods improve throughput and reduce errors associated with sample storage, processing, and complexity, low analyte concentrations and harsh environment conditions limit their accuracy and suitability.^19–23^ Therefore, fast, durable, and portable sensory platforms for detection and isolation of desired compounds from environmental samples are required to exhibit advantages over existing analytical techniques.^24–26^ These new platforms will pave the way towards an outstanding sensitive and selective performance that will undoubtedly advance future developments in food safety, environmental monitoring, and healthcare fields.^2,10,27,28,146,147,152^

**Fig. 1:**
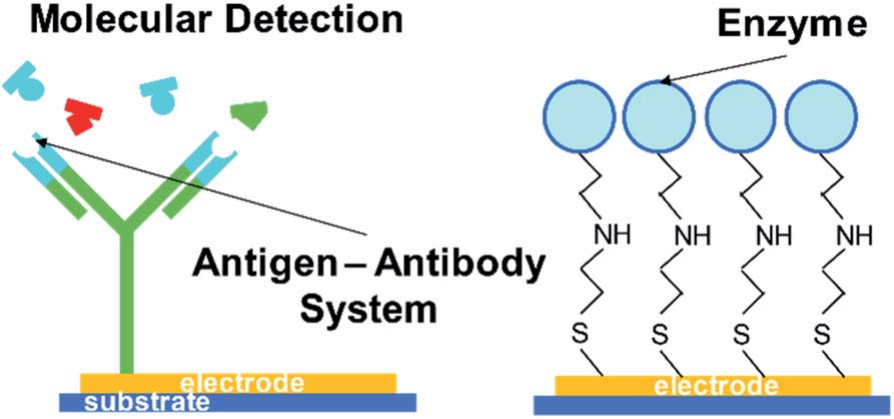
Schematic representation of hybrid devices, (left) antigen anchored to an electrode for selective sensing; (right) enzyme anchored to an electrode for specific catalysis and sensing.

**Fig. 2:**
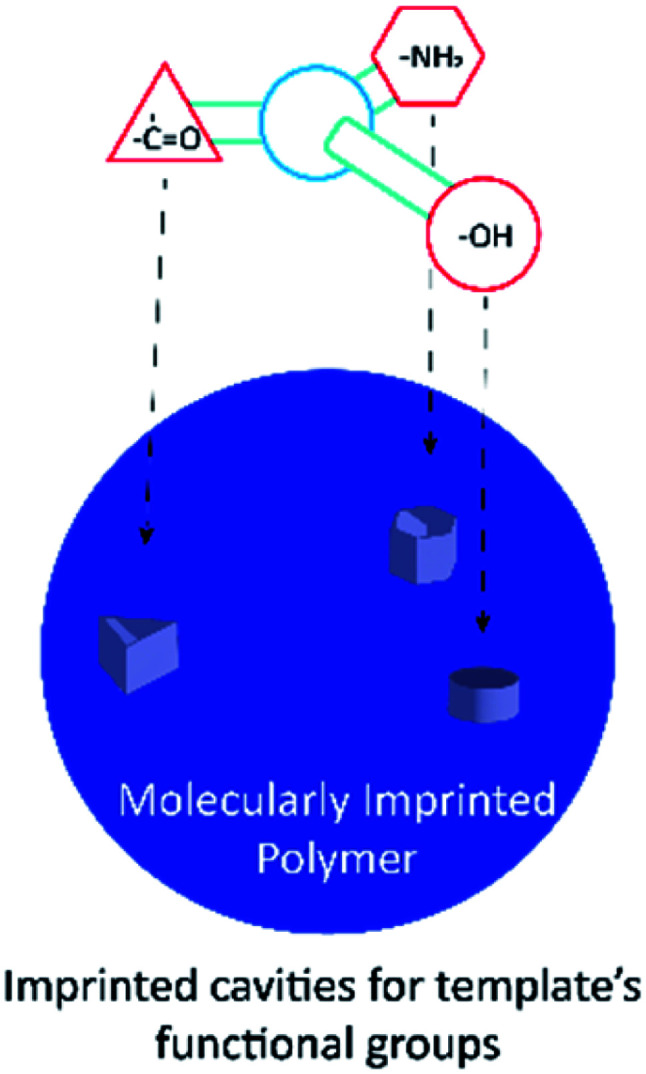
Molecularly imprinted polymer scheme, particle with imprinted sites for the template molecule.

One possible way to create such a novel sensing platform is to take advantage of biomolecules attached in electronic devices (see Fig. 1 for the schematic illustration). Its molecular recognition capability, sensitivity, and dimensions create an attractive hybrid solution that combines nature's performance and selectivity as a signal transducer with electronics^99^ that are used and commercialized as a sensor.^30–38^ However, the main challenge with this architecture is that it stills rely upon physiological conditions such as pH and temperature. A solution to overcome this challenge has been to use the molecularly imprinted polymer (MIP) technique, which mimics the key-lock principle with synthetic molecular structures.^23,25,35,38–44^ This technique relies upon the creation of specific size, orientation, and functional cavities for a template (Fig. 2) whose binding event can be transduced by a chemical or physicochemical event.^45–47^ The application of tailor-designed imprinted polymers in analytical chemistry needs to be tightly controlled since the high value applications of functional polymers in analytical science generally require well-defined interfaces, including those in precisely synthesized molecular architectures and compositions.^17,38,47–49^

In the past few years, molecular imprinting has been considered as a promising technique that allows the creation of synthetic receptors, consisting of highly crosslinked porous rich materials with recognition properties comparable to the biological systems related to active sites or cavities with shape, size, and functional groups for a specific target molecule (also known as template).^22,50,51^ Another important property of these synthetic sensing materials (artificial receptors) is that they serve as one of the most attractive components of chemical biosensors because of their long term stability and low production cost compared to the synthesis and extraction of biological receptors such as antibodies and enzymes.^52,53^ The last mentioned was the inspiration for this technique creation as a route for polymer design and its use for compound isolation in the solid phase and electrochemical sensing applications.^3,22,25,54,55^

The first paper that specifically reported an “imprinted polymer” appeared in 1984, written by K. Mosbach and B. Sellergren,^39^ although another paper entitled “Enzyme-Analog Built Polymers” was written in 1973 by Wulff. These early researchers developed several elements of MIP.^56,57^ The Mosbach group focused on the development of noncovalent interactions between the functional polymer and target molecule also known as a template, while Wulff tended to research covalent binding to imprint polymers.^45^ The difference between these two methods is dictated by the chemistry methodology necessary to attach and remove the template from the MIP.^42,45,58–61^ Certainly, the covalent route is expected to produce a more homogeneous distribution of cavities and, potentially, more target specific MIP.^62^ As mentioned before, several authors described the development of separation and sensing materials, and Wulff and Shea extensively employed imprinted polymers in catalytic reactions, ranging from biochemical to biomedical applications.^49,63,64^ These applications laid the foundation for specific sensing platforms since the term “molecularly imprinted polymer sensor” started to appear by S. Piletsky in 1992 for the first time.^65–68^ This polymer methodology has attained great popularity due to its versatility and applicability; its applications have been found in catalysis, separation, filtration, and quantification. These can be adapted to several transducing systems, such as electrodes, transistors, and even the coupling to instrumental techniques for its specific binding capacity.^53,65^

This review attempts to condense the fundamental concepts, synthesis methods, and performance relating to nanostructured organic/inorganic chemical sensors based on MIP *via* the controlled/living radical polymerization (CRP) method of reversible addition–fragmentation chain-transfer synthesis (RAFT) and their application as selective receptors for environmental monitoring. With this specific focus, this work seeks to elucidate the potential of the polymerization technique due to their superior recognition capacity, stability and versatility. It ties the individual and segmented information of our literary research into a concise text that enlightens on the novel solution that RAFT synthesis technology for MIP offers for receptor sensing technologies. This work offers fundamental insight for the scientific community, readily capturing the advantages and characteristics of various classes of sensor nanomaterials in one place, along with their synthesis conditions, and integration towards smart synthetic materials capable of specific analyte monitoring and sensing applications.

## 2. Applications of imprinted polymers

Designing and constructing functional polymers is recognized as one of the most effective routes for analytical science applications such as chromatographic separation, solid-phase extraction, fluorescent or colorimetric sensing, just to list a few.^23,38,69–71^ The critical role of the tailor designed polymer is the capability to selectively interact with the target molecule, reducing its function as a selective adsorbent/receptor on its functional cavities.^61,72,73^ The capacity to design cavities with specific morphology, orientation, and functional groups has been exploited for different purposes such as extraction, synthesis, and catalysis.^3,45,50,60,69,74–76^ Later development and improvement of these receptor's capabilities opened up the opportunity to quantify these binding events with the combination of physicochemical methods.^4,71,77^ This led to the implementation of these polymers as stimulus transducing elements in sensorial platforms.^78,79,99^ These platforms range from biosensors for biomedical devices to food security and environmental monitoring in quality processes for water, soil, and air, toxic residual removal materials, smart/responsive structures to stimuli (thermic, magnetic and optical).^29,41,65,68,80–83,143–145,149,150^

The aforementioned applications demand the synthesis of these polymers with controlled molecular structures and specific surface topologies to achieve the intended binding capacity.^42,61^ Normally, free radical bulk polymerization has been used as an effective approach for both the commercial and lab scale production of MIP because of its compatibility to a variety of monomers, mild polymerization conditions, and tolerance to many different solvents and impurities during the reaction.^48,84–87^ However, the main drawback is a poorly controlled polymerization process because of the fast propagation and inevitable radical termination in a variable chain length.^17,88,89^ Mentioning these, a series of previous works were summarized in Table S1† to identify a relationship between the research approach and the analytical application of the MIP in environmental and biological monitoring. It is found that most MIP literature studies focus on a specific application and consider an empirical approach for the design of the material.^39^ Also, a series of reviews highlight that using the living/controlled polymerization technique is the most accepted alternative to improve the MIP features such as selectivity, sensitivity, and maximum adsorption capacity due to the achieved molecular structure.^43,69^ Nonetheless, the topic is viewed from an empirical point of study since most of the works focus on the performance of the developed polymer without comparing the used polymer design fundamentals. This situation is visible in the applications column in Table S1.† Since the reported reviews analyse the MIP in a specific field of application, regarding its performance, they select those with clearly superior capabilities among the other works.^19,22,24,28,30,51,90^ To support this analysis, a scientific basis of polymer science could set a combination for the rational design of these polymeric receptors as the empirical performance is being compared. This situation opens the opportunity to profoundly understand how to design an MIP for each specific industry since its design depends on a series of experimental parameters, processes, and environmental conditions.^25,43,73,91–93,144,151^

On the other hand, there are a few works that combine computational chemistry to identify the most stable active site between the functional monomer and the template.^42^ This was taken as an opportunity to start analyzing the parameters to tailor design these polymeric receptors for sensing in analytical platforms, focusing on *living* polymerization technique effects over the imprinted cavities.

## 3. Molecularly imprinted polymers synthesis as a strategy for selective and durable sensing

Several methods have been established for the synthesis of MIP; Ndunda *et al.* stated in 2020 that these can be roughly divided into covalent, noncovalent, and semi covalent imprintings.^47^ On the other hand, there are several production methods concerning different host–guest binding properties.^30,94^ These methods rely on the presence of the template during synthesis which can follow several strategies for later remove exceeding monomer and solvent from the reaction mixture such as phase inversion using polymer precipitation by addition of an incompatible solvent or by evaporating the solvent from a networked solution of polymer plus template, and soft lithography or surface stamping.^29,148^ “Table 1 summarizes a series of applications for these production methods, showing that the synthesis method does not dictates the application but the template/monomer interactions. The production method relies upon the available infrastructure and material's expected binding capacity and performance since each methodology results in a different imprinting effect.” The present review focuses on this synthesis methodology since it shows advantages on energy consumption and requires simple laboratory equipment in comparison with other reported methods.

**Table 1:** Summary of MIP applications and their production method, modified from (ref. 50, 90 and 95)

MIP production method	Applications	Reference
Phase inversion	Food security	30
	Essential oils extraction	14
	Bio catalysis	44
Ground bulk	Biomarker's monitoring	15 and 43
	Filtration membrane	47–49
	Soil quality sensing	29, 45 and 46
Soft lithography	Wastewater monitoring	43 and 50
	Biosensors	51
	Biofluid analysis	52

MIP synthesis includes several polymerization methods such as ground bulk which generates irregular size and shape particles; precipitation improves this situation with no control over molecular weight distribution; emulsion increases monodispersity, iniferter for a fine-tuned reaction advancement, and core–shell for smart/responsive particles.^44,87,95,147,148,152^

Unfortunately, the MIP beads/particles enlisted by the above methods exhibit limitations when prepared through a free radical polymerization, including the fact that active recognition sites embed deeply in the particle require long diffusion time, poor mass transfer, complex and non-scalable manufacturing, and bad regeneration performance, greatly reducing their application options.^3,66,96–98^ A comparison between these imprinting methods is discussed in Table 2.^3,96^

**Table 2:** Summary of MIP comparison of different free radical imprinting methods of MIP. Modified and extracted from ref. 44, 90, 93 and 148

Imprinting method	Advantage	Disadvantage
Bulk	Simple and fast preparation; no instrumentation required; high purity polymer	Sieving required to achieve small particle size; irregular particle shape and size
Suspension	One step polymerization process; spherical particles	Big particle size (hundred micrometres); poor recognition activity
Emulsion	High yield; water soluble	Remnants of surfactants; low imprinting
Seed	Spherical particles; high monodispersity	Time consuming process
Precipitation	One step preparation; uniform, and spherical particles	Excess of template; high dilution factor

In the other hand, it is important to consider that this absorption property can be used as a key element when we are talking about pollutant removal from environment, the works from Foroughirad, *et al.* are a clear example of an holistic approach when talking about environmental applications since these materials can be designed to sense and remove pollutants from environment.^141–143^

**Fig. 3:**
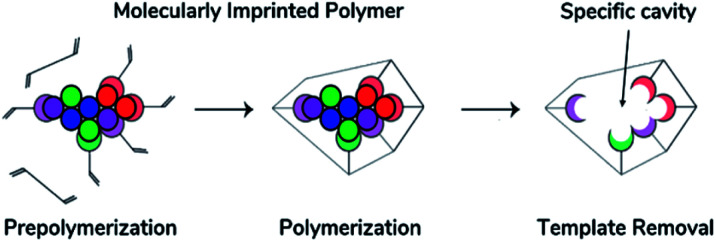
General imprinting technique process (left to right): prepolymerization, polymerization and template detachment. Modified from Belbruno, J., *J. Chem. Rev.*, **119**, 94–119 (2019). Copyright 2017 American Chemical Society.^29^

The synthesis procedure of MIP generally includes three steps shown in Fig. 3. As mentioned above, a template-monomer complex is first formed *via* noncovalent or covalent interactions between the template molecules and functional monomers containing vinyl groups which are crucial for radical polymerization.^93^ The interaction between these two depends on the selected monomer and solvent nature. Although there are reported tables for the selection of these compounds depending on the intended interface with the template, the literature has shown that the imprinting method plays a key role in the performance of the material.^26,38,51,65,89,100,101^ An analysis of the advantages and disadvantages of free radical polymerization is shown in Table 2.^38^

The second step in MIP synthesis requires the presence of the template-monomer complex which was previously prepared. If necessary, it can be added an assistant material to achieve a porous rich polymer and enhance mass transfer, and consequently, a crosslinker agent is incorporated in the reaction mixture to create a network of these complexes. Afterward, an initiator starts the polymerization reaction, and the receptor is obtained *via* free radical propagation which is mostly induced by heating or UV-light initiation.^30,42,44,46,61,73,103,104^

Finally, the template molecules are removed from the polymer *via* chemical etching, leaving the imprinted cavities as binding sites in the particle, which fit the template in dimension, morphology, and functional groups,^51,66,68,104,105^ resulting in highly selective receptors for the template or its analogues. The achieved binding selectivity is comparable to a natural antibody or enzyme, resulting in the combination of nature's sensitivity and analytical performance at a low cost and ease of preparation with a polymer's high thermal and chemical stability, and excellent reusability.^30,44,61,97,103,106,107^

The development of a biomimetic artificial biosensor can still be improved by engineering processes enhancing the driving forces of molecular recognition by the integration of complementary steps to control the reaction progress and tailor-design the polymer architecture and molecular weight. These properties can be tuned by the used initiation method, and the most reported technique to regulate free radical polymerization is the implementation of a controlled/living radical polymerization technique, which regulates the termination step and limits the side reactions until polymerization is achieved.

## 4. Controlled radical polymerization application in analytical polymeric interfaces

Controlled/living radical polymerization (CRP) has revolutionized the field of polymer science since the 1990s. A great focus has been put on its industrial origins, leaving aside the fundamentals, and achieving highly specialized properties instead of being a commodity nowadays.^46,69,102,109,110^ This polymerization method shows great performance in terms of polymerization degree and control over the polymer architecture. It can considerably improve the homogeneity of the crosslinked structure of MIP particles and elucidate their structure property relationship.^11,28,69^ Therefore, its versatility has made the CRP based techniques the most popular in the preparation of various advanced functional polymers.^89^ For analytical chemistry, diverse polymers with controlled architectures including homopolymers, block copolymers, molecularly imprinted copolymers, and grafted copolymers were synthesized by CRPs for target isolation or sensing.^46,111^ In this review, we present an overview of the recently developed advanced functional interfaces by a specific CRP approach in analytical science applications.^45,110^

### 4.1. Types of controlled/living radical polymerization

The definition of controlled/living polymerization is a polymerization reaction, where the polymerization takes place in a living way. Here in this process, the side reactions (such as termination and transfer reactions) are negligibly small, and the polymerization degrees of the resulting polymers increase linearly with the monomer conversions.^23,43,89^ In recent years, the most extensively investigated CRPs included atom transfer radical polymerization (ATRP), reversible addition–fragmentation chain transfer (RAFT) polymerization, and iniferter induced “living” radical polymerization.^23,43,69,71,77,111–115^

**Fig. 4:**
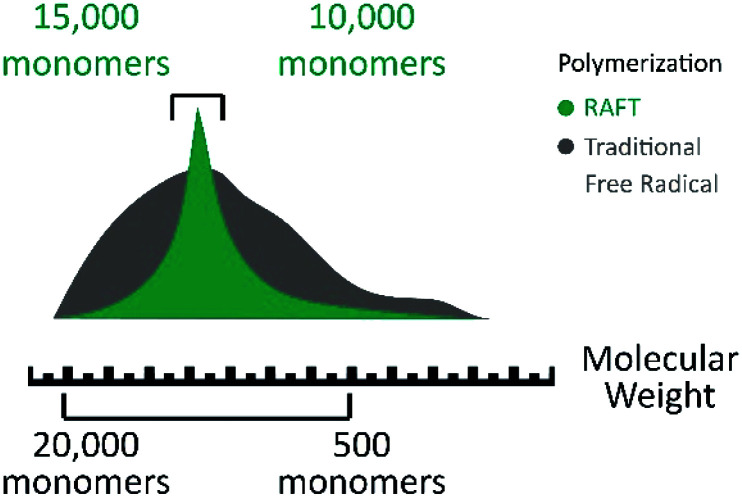
Molecular weight dispersion comparison between controlled/living radical polymerization (green) and radical polymerization (gray). Modified from Sigma Aldrich Copyright, RAFT polymerization products.

All these CRP techniques rely on the concept that the reactive growing radical species is transiently and reversely converted into the dormant state *via* the formation of the covalent terminal.^88^ This ability to activate and deactivate the radical state lets the reaction advance at a controlled rate, so most of the monomers react and the generated chains present a similar length, which is verified by analyzing the molecular weight of the synthesized polymer (Fig. 4).^69,79^ This is verified in the reported literature, which also evidences that ATRP is more suitable for films with a specific thickness, and it is not suitable for industrial production because it depends on a metallic catalyst and the related and indirect costs rise.^69,111,116^ On the other hand, RAFT polymerization shows an advantage for large scale production and offers a simple methodology. It does not require a catalyst or specific ambient conditions in comparison with ATRP which is highly sensitive to the presence of oxygen in the reaction mixture.^108,116^

#### 4.1.1. Atom transfer radical polymerization (ATRP)

**Fig. 5:**
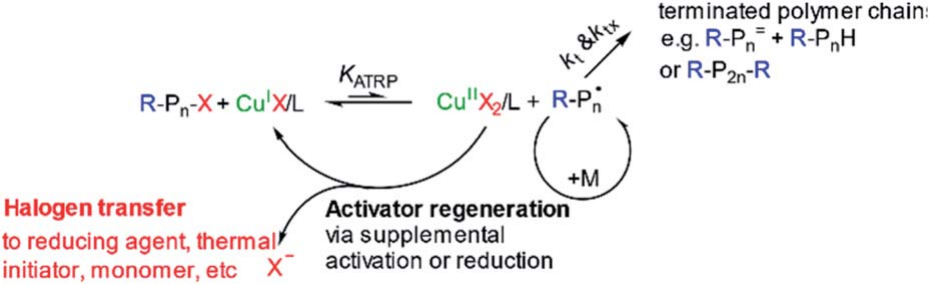
Mechanism of ATRP copper-mediated polymerization. Pn: polymer; M: metal catalyst; X: halogen, R: radical. Reprinted from Wang *et al.*, 2016, *Journal of Applied Materials and Interfaces*.^69^

ATRP is the most studied technique due to its specific features, including the vast options of initiators, a broad scope of compatible monomers, and mild reaction conditions.^112^ This is a catalytic process that is mediated by diverse redox active transition metal complexes. As shown in Fig. 5, the process involves the transfer of a halogen atom (X) from the dormant species (for example, alkyl halides (R–X) or polymers containing “living” halide final-groups (Pn–X)) to a low oxidation state metal catalyst (Mt–L; Mt stands for transition metal species, L for ligand), yielding a free radical (R· or Pn·). For this technique, the initiator requires a halogen (X = Br/Cl) and a functional group that can stabilize the formed radical (for example, cyano, carboxyl, phenyl). The catalyst metal complex (Mt–L) establishes a reversible equilibrium between growing radicals (Pn·) and dormant species (Pn–X). This technique has some drawback for industrialization since the halide aiding as the initiator is poisonous and the reduced metal catalyst is too sensitive to oxygen. Thus, using this technique in large scale systems would need to consider an environmental risk of contamination and the fact that the necessary equipment may be too expensive to limit the oxygen concentration in the polymerization vessel.^89,117^

#### 4.1.2. Reversible addition–fragmentation chain transfer polymerization fundamentals

**Fig. 6:**
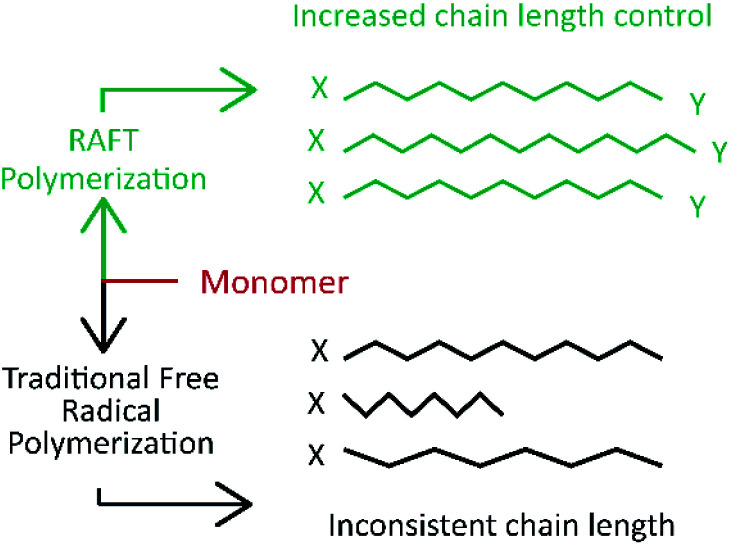
Polymer chain length comparison between RAFT polymerization (green) and traditional free radical polymerization (gray). Modified from Sigma Aldrich Copyright, RAFT polymerization products.

On the other hand, RAFT polymerization is a versatile controlled/living radical technique, which is quite effective in solution and bulk polymerization systems.^79^ The RAFT polymerization comprises an addition–fragmentation equilibrium by using thiocarbonylthio compounds as chain transfer agents (CTA) to regulate radical polymerization advancement.^43,109^ This polymerization is initiated by a conventional free radical initiator (I), where the homolytic cleavage results in two reactive main free radicals (I·/Pn·).^88^ The controlled/living character of the RAFT polymerization is given by the equilibrium among the polymeric radicals (Pn·), the dormant species, and intermediate radicals. This polymerization can be used to synthesize well defined polymers for numerous monomers with a low polydispersity index (Fig. 6) under mild reaction conditions.^43^ This can result in the increase of chain length control along with the synthesis. The main advantage regarding industrial processing is that this CRP technique can be employed in several types of free radical polymerization such as solution, emulsion, and suspension polymerizations since it does not depend on a catalyst or specific reaction atmosphere.^73,86,87,89^

**Fig. 7:**
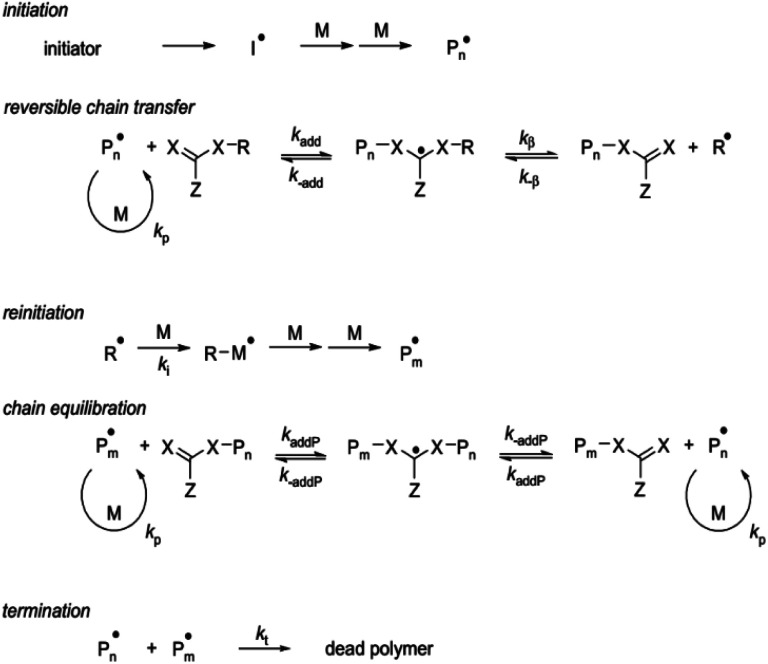
Mechanism of RAFT polymerization. Reproduced with permission of the American Chemical Society, reprinted from Wang *et al*., 2016, *Journal of Applied Materials and Interfaces*.^69^

In addition to this, RAFT polymerization has shown to be one of the most functional and adjustable methods for bestowing living attributes to radical polymerization. It provides reversible deactivation of propagating radicals by degenerate chain transfer as shown in Fig. 7. The chain transfer step has been designated as degenerate since the process involves an exchange of functionality between the chemical species.^97,108,118^

The mechanism consists of an activation/deactivation that degenerates a chain transfer as shown in Fig. S1.† Primary radical binds with the CTA and creates polymeric CTAs *via* consecutive addition of monomers to its thiocarbonyl double bond. Polymeric CTA is converted into this state by intermediate reaction reversibly. This reaction is based on a chain equilibrium in which free radicals are not formed or destroyed. The reaction is regulated so that the concentration of initiator derived chains is negligible and only primary radicals subsist in previous steps of the reaction.^118,119^

Different authors have reported the use of the RAFT technique as a route to improve MIP active sites (Table S2†) in terms of homogeneity and sorption efficiency.^120,121,144^ This sets the path toward a study to determine the variables that can be tuned to design this functional polymer. As mentioned in the previous section, the selection of an imprinting technique is a setpoint for artificial receptor design since this implies a series of properties, needs, and performance. The selected imprinting method for this research focuses on radical polymerization in consequence of its promptitude and no requirement for sophisticated instrumentation.^122,123^ However, the process design does not end at this point; the selection of a specific monomer consists of identifying compatibility with the CRP type and the intended application as shown in Table S3† where a series of works in the use of RAFT-MIP were summarized. This analysis aids in the selection of the CRP technique depending on the template-monomer generated complex conceived as the active site for the MIP.

## 5. Reversible addition–fragmentation chain transfer polymerization used in the design of molecularly imprinted polymers

The RAFT mechanism precisely controls molecular weight, dispersity, end groups, and architecture of synthesized polymers. Regarding the architecture, preestablished structures and topologies are available such as stars, combs, and particles; this variety leads us to design the polymer particle structure just by selecting the RAFT agent.^58,96,113,119^ The versatility of this polymerization technique relies upon the use of “living” core polymer microspheres and surface mediated polymerization for the controlled growth of uniform MIP layers with adjustable thickness.^43,102,122^ The control over these properties provides the opportunity to design different advanced polymers with high efficiency and several applications. The resulting materials have superior properties compared to a traditional free radical polymerization. An example of this is the presence of homogeneous structures which produce higher affinity and an efficient mass transfer phenomenon while achieving a water compatible surface. Additionally, the RAFT mechanism can adjust the effect of decreasing binding ability produced from the reduction of apparent crosslinking degree. The higher improvement in morphology *via* RAFT polymerization is originated from more homogeneous distribution of crosslink points in the MIP structure.^124^ This broadens the application scope and performance for MIP since water compatibility is necessary for environmental monitoring. Otherwise, the material won't interact with the analytes and its performance will be affected.^114,122^ The following section enlists several characterization techniques for the evaluation of the polymer performance.

## 6. Determination of RAFT-MIP materials performance

**Fig. 8:**
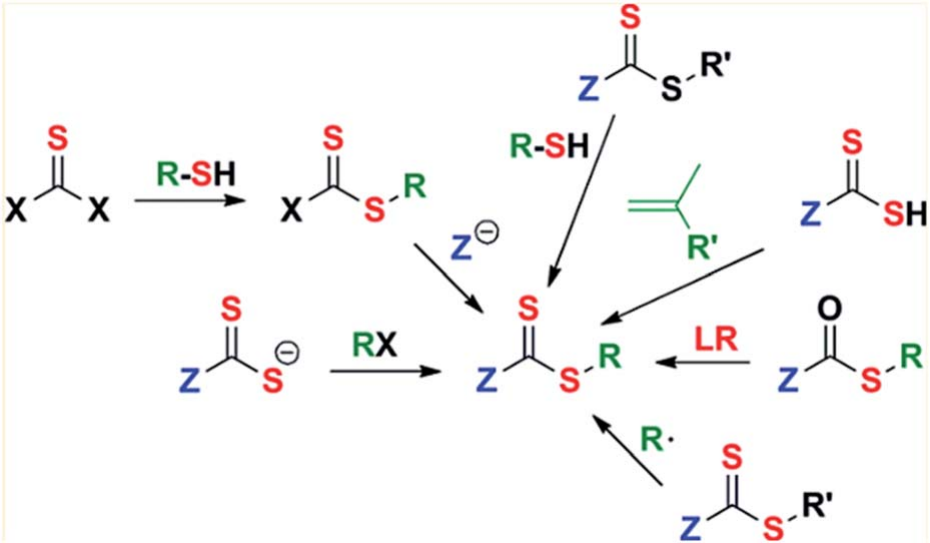
Examples of RAFT chain-transfer equilibrium possibilities. Modified from Zhang, H. *et al*., *Eur. Polym. J.*, **49**, 579–600 (2013).^122^

The generation of predetermined molecular weight and narrow molecular weight distributions, as mentioned before, results in more homogeneous polymer structures, hence, improving the imprinting effect and availability of the specific cavities for the template.^45,47,61^ Reactive terminal groups can be purposely manipulated to build additional functionality in the polymer backbone architecture including graft, star, and gradient structures.^69,114^ Achieving an optimal control in RAFT polymerization requires choosing an appropriate RAFT agent for the monomer to be polymerized and the reaction conditions. As shown in Fig. 8, the Z and R groups both play critical roles in determining the outcome of polymerization by participating in the chain transfer equilibrium. Its role is to determine the rate of addition and fragmentation, and they control the efficiency of chain transfer and the likelihood of retardation or inhibition of the radical state.^77^ Once this reaction design is made to achieve a specific molecular architecture and terminal functional group *via* a specific RAFT chain-transfer equilibrium, the resulting polymer needs to be evaluated so the efficiency and accuracy of the tailor made material are evaluated.^125^ It is important to evaluate the following properties because they are expected to be improved since RAFT was added in the reaction design despite the characterization technique is the same for both free radical and controlled radical polymerizations.

### 6.1. Dispersity and polymerization degree

**Fig. 9:**
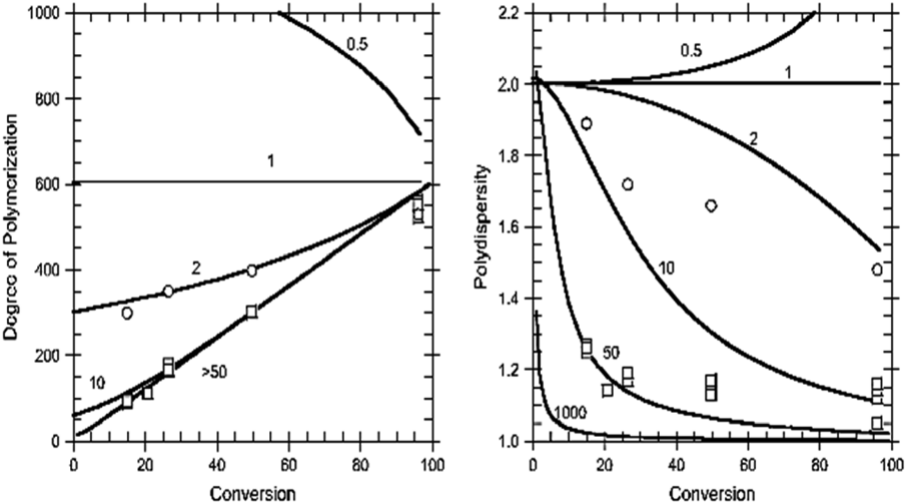
Degree of polymerization characterization of RAFT-MIP. (left) Degree of polymerization in relation to the conversion of monomer: RAFT agent; (right) disparity of the resulting polymer and its *Đ* value. Reprinted from Gody, G., *et al.*, *Macromolecules*, **47**, 639–649 (2014).^126^

The predicted dependence of the degree of polymerization and dispersity of the polymer formed on monomer conversion and the transfer coefficient (*C*_tr_ = *C*_−tr_) for an ideal polymerization (no termination) with reversible chain transfer is shown in Fig. 9. The predicted degree of polymerization is simply the ratio of [monomer consumed]: [RAFT agent consumed]. The characteristics often associated with living polymerization, namely, the straight-line dependence of molar mass on conversion (and a low dispersity, *Đ* < 1.2), require a *C*_tr_ of at least 10. The most effective RAFT agents have a *C*_tr_ > 100.^79,119^

The degree of polymerization is determined by the ratio of the concentration of consuming monomer to the sum of the concentrations of consuming transfer agent and decomposed initiator.^113,119^ Dispersity depends on the ratio of the rate constants of exchange reaction propagation. If the number of new chains formed by the decomposition of the initiator is much lower than that produced from the transfer agent, the rate of transfer is fast, and RAFT can control polymer chain growth by controlling the overall reaction rate.^118,120^ The RAFT agents should be carefully chosen for a specific polymerization system. Various dithioesters, dithiocarbamates, trithiocarbonates, and xanthates have been effectively used as CTAs to control the polymerization process.^126^ To efficiently evaluate this parameter, the Gel permeation chromatography (GPC), also known as size exclusion chromatography (SEC), is used for Molecular weight (*M*_w_) determination of polymers, which is based on size exclusion phenomena.^23^ This technique provides the *M*_w_ and the molecular number, requiring calibration with polymers of known *M*_w_, such as polyester standards. An example of this validation is the work done by Cai *et al.* (2019); they combined RAFT-MIP initiated by photo-induced electron transfer method to develop an electrochemiluminescence sensing platform for melamine quantification.^109^

### 6.2. Polymerization evaluation with Fourier transform infrared spectroscopy

Fourier transform infrared spectroscopy (FTIR) analysis aims to distinguish and compare functional groups of (1) the monomer, (2) imprinted polymer, and (3) non imprinted polymer (NIP) through the incidence of infrared radiation on the sample and consequential analysis of the generated interferences in the spectrum. When the sample interacts with the radiation, each functional group of the sample generates specific adsorption bands in the spectrum obtained. The observation that only spectra with the bands of the template monomer and the crosslinking agent appear in both the imprinted polymer and the blank or NIP verifies that the ion does not interfere with the polymerization and that the interaction is of a non-covalent type. The work done by Alahi *et al.* used FTIR to evaluate the type of binding between the template and the monomer to develop an ion imprinted polymer, depicts that the achieved interaction was non covalent since the template's IR band does not appear in the MIP or the NIP.^20^

The resulting spectra provide information about the presence or absence of a functional group and this allows monitoring of the polymerization stage and is considered as a confirmation analysis of the template binding on the imprinted sites.^42,77,107,127^

### 6.3. Adsorption analysis with UV-vis

To characterize the polymerization efficiency as imprinting technique, the polymer's capacity as sorbent, and its viability of use in several applications, it is necessary to execute an adsorption kinetics study of the MIP, the NIP, and the target by placing them in a stock solution of the template. This procedure evidences the template milligrams per MIP gram adsorbed at room temperature, thus obtaining the maximum adsorption capacity and the evaluation of the intended cavities by also evaluating the blank polymer (NIP).^11,91,116,128^ On this scenario by Ishak, *et al.* the template solution was analysed with a radiation source at a wavelength of 200–205 nm corresponding to nitrate with UV-visible spectroscopy.^38^ In this way the concentration changes in the stock solution over time will be obtained by the attribution of the adsorption of these ions in the polymer. It is important to consider the decrease in volume generated by the aliquots to correct the concentration of the solution as the analysis evolves.^39^ Previous works have shown that the adsorption capacity increases for the imprinted polymer due to the generation of specific cavities; this situation is observed where the NIP presents a lower adsorption capacity.^26,30,129^

## 7. RAFT-MIP applications in analytical sensing

Chemical versatility and mild reaction conditions of the RAFT mechanism make it compatible with MIP synthesis. The combination of these two achieves a homogeneous polymer network which is traduced as more accessible cavities/receptors for the template. When implemented as a transducing element in sensors, sensitivity and selectivity are improved. The most common sensor platform used in this field of analytical science is the Interdigitated Electrode (IDE) because of its simplicity of design, fabrication, and versatility to be combined with functional materials.^48,130^

**Fig. 10:**
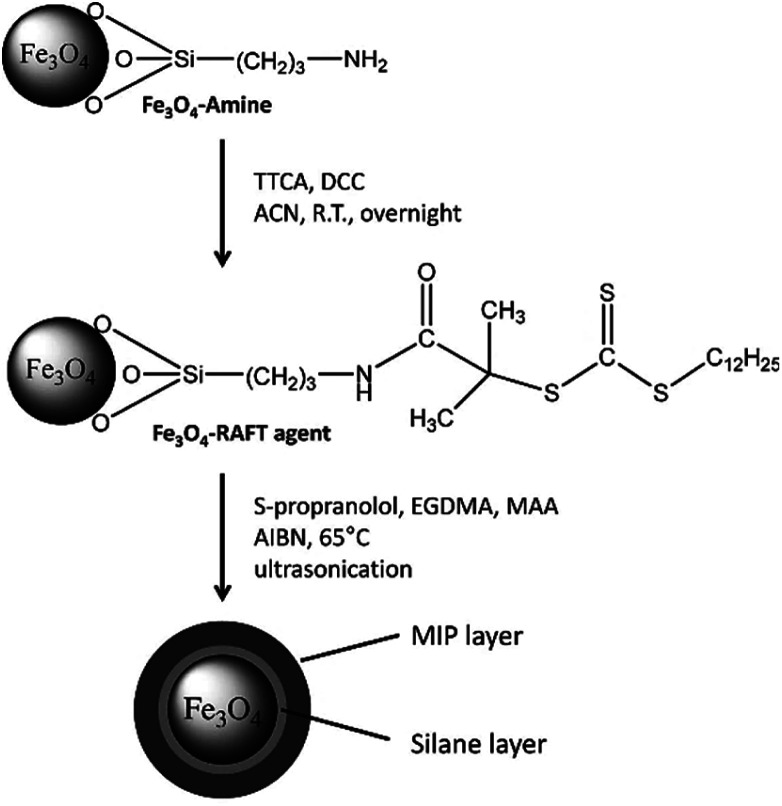
Reaction scheme of the surface modification of amino-functionalized Fe_3_O_4_ nanoparticles and the subsequent grafting of a MIP layer. Reprinted from Gonzato, C., *et al.*, *Adv. Funct. Mater.*, **21**, 3947–3953 (2011).^109^

As mentioned before, the “living” mechanism of RAFT, slow polymeric chain growth, and the negligible chain termination decrease the effects of irregular imprinting, the low binding capacity, and slow mass transfer of traditional MIP synthesis. So, the potential use of the RAFT mechanism is an improved route to tailor design the molecularly imprinting technology process. On the other hand, RAFT has shown to be a versatile polymerization technique because of the several molecular architectures that can be produced. Among the previously mentioned ones, the molecular brush is the most promising structure since the proposed methodology consists of anchoring the RAFT CTA in the surface of a particle or substrate, so the polymer branch elongates attached to the substrate which is intended to play a critical role in the design of sensor devices (Fig. 10). The critical point here is the polymer deposition or integration to the sensor because depending on the synthesis method, the produced MIP's properties would be affected during and after deposition in the device. To tackle this challenge, polymer brushes are the favourite option because the *in situ* polymerization solves the deposition stage, and a more efficient interface between the polymer and the sensory platform is developed. This is because the traditional method of deposition of the MIP particles in the IDE does not ensure the interaction between the polymeric and the metallic interfaces so the binding event will not be correctly transduced in an electrical signal.^131,132^

Several authors have reported the use of surface initiated RAFT-MIP synthesis to improve the interface between the MIP and the substrate. One example is a work by Gonzato *et al.*; they designed a core–shell particle by anchoring the RAFT agent in a Fe_3_O_4_ amine functionalized particle's surface. By doing this, the polymer chain adopted a brush like structure around the particle, so it formed a layer. The ending groups of the polymer were intended to be used as anchors to fine tune the particle's surface properties so a responsive/smart material could be developed. This property is desirable and versatile because the opportunity to grant specific properties into each layer of a particle or surface sets a new path of developing advanced and stimuli responsive materials. Its behaviour can be “programmed” by understanding and controlling the used polymerization technique.^30,75,102^

## 8. Experimental practicalities

The understanding of the polymerization technique starts by defining the monomers used in RAFT polymerization. These could be divided into two groups, namely the more active monomers (MAM) and the less active monomers (LAM).^133,134^ The former has a double bond conjugated with an aromatic ring such as styrene or 4-vinyl pyridine, a carbonyl group such as acrylamide, acrylic acid, or other acrylic monomers, or a nitrile such as acrylonitrile. In contrast, LAM has a double bond adjacent to an oxygen (vinyl acetate), nitrogen (*N*-vinylpyrrolidone), halogen (vinyl chloride), or sulphur ((4-bromophenyl) (vinyl)sulfane) lone pairs or saturated carbons.^124^ MAM are typically polymerized with Di thiobenzoate or trithiocarbonates RAFT agents, while less active CTAs are used for highly reactive propagating radicals provided by LAMs.^135^ This sets the first step to designing a MIP by implementing the RAFT mechanism because previous works by Alahi and Sellergren separately claim that the greater affinity between the monomer and the template sets the difference on the polymer's performance such as its binding site accessibility, and capacity shown. In addition to this, the result of this tailor made reaction against the FRP is that the traditional method produces polymers with a wide distribution of pores and low swelling tendency, with many poorly accessible binding sites, which results in a lower sorption capacity.^30,56^

**Fig. 11:**
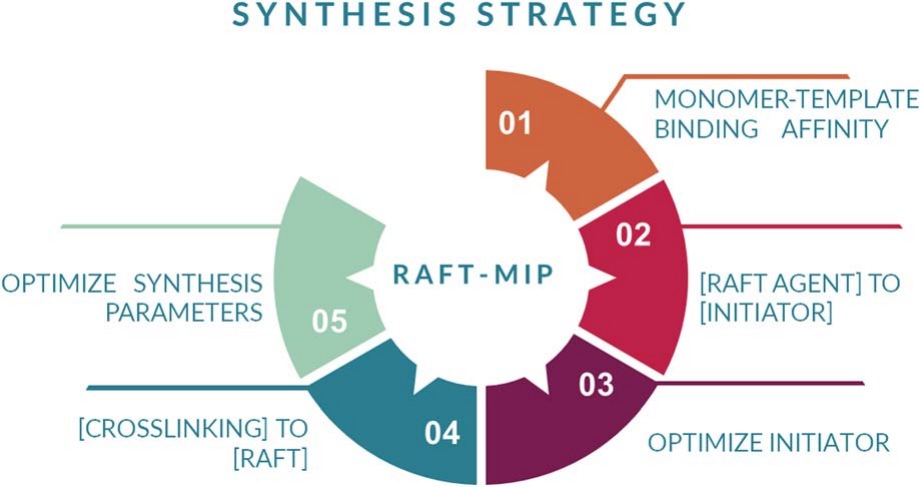
Identified steps for RAFT-MIP synthesis strategy.

A series of steps are identified to synthesize a MIP by RAFT mechanism, as shown in Fig. 11. It starts by selecting the monomer–template complex with higher non covalent affinity.^126^ Then, the compatible RAFT agent and initiator with the intended monomer are identified, and the initiator concentration is optimized to increase the propagation rate and the fraction of living chains while achieving complete monomer conversion by selecting a monomer with high propagation rate coefficient and a RAFT agent with low retardation. The relationship of these parameters is shown in eqn (1). Next, the crosslinking agent is selected by maintaining a higher relationship with the RAFT agent concentration, so the generated material achieves a specific mechanical strength. Finally, synthesis temperature (when using heat in the propagation stage) and mixing speed are optimized according to the mixture viscosity.^90,136^1
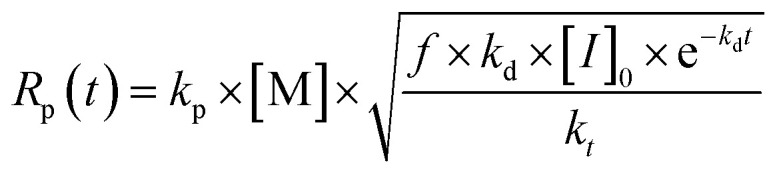


Polymerization rate in the RAFT process where *R*_p_ is the polymerization rate, *k*_p_ the propagation rate coefficient, [M] the monomer concentration, *f* the initiator efficiency, *k*_d_ the decomposition rate coefficient of the initiator, [*I*]_0_ the initial initiator concentration, and *k*_*t*_ the termination rate coefficient.

## 9. Challenges and opportunities

Research opportunities in the field of MIP and the properties of this class of sensors include environmental safety, excellent sensitivity, selectivity, and stability.^30,74^ In this case there is a broad range of capabilities but the key feature of formulating MIP is the opportunity to tailor responses to a specific need.^81^ The relevance of this relies on the understanding of the compatibility of formulation components and the polymer's aging mechanisms. Nonetheless, many challenges affect the performance of polymers such as template leakage, low binding capacity, slow mass transfer, heterogeneity of binding sites, incompatibility with aqueous media, and hydrophilic compound imprinting and biological macromolecules.^3,124^ Nowadays, the main challenge of using polymers in molecular recognition is the lack of control over the polymer properties. This limitation is originated in part from the polymerization method. So, the opportunity relies upon the efficient design of the CRP for the selected need.

## 10. Conclusion

The evolution of smart synthetic materials that mimic the natural ligand-receptor binding has experienced a quick expansion in the last years and controlled radical polymerization (CRP) has undoubtedly contributed to this result. Although free radical polymerization (FRP) is still the main approach for molecularly imprinted polymer (MIP) synthesis, the intrinsic benefits of CRP make it more attractive for imprinting. Consequently, in recent years, more and more researchers are replacing traditional FRP with CRP techniques, and this trend will likely continue in the following years, making CRP the main polymerization approach for imprinting. CRP techniques have allowed better control over the MIP architectures, obtaining more homogeneous polymer networks that present a greater homogeneity of the binding site.^69,122^ The materials developed under these conditions have improved kinetics, resulting in imprinted polymers with a better overall performance in terms of binding affinity and specificity. Much works still need to be done concerning ‘plastic’ or ‘synthetic’ antibodies, particularly in the context of their synthetic strategies.^124^ Further research still should be targeted to obtain higher imprinting yields with a more homogeneous size distribution capable of being produced on a large scale. By achieving the development of the proposed solution, a whole process would be required to expand the application of the designed polymeric films since several industries require a specific interaction for separation, extraction, and sensing.

To this end, the integration of the Internet of Things (IoT) with advanced functional materials offers the great possibilities for environmental monitoring.^130^ Nowadays, several companies have developed technology for real time quantification and detection of specific chemical species. To create this specific interaction, they use ion selective electrodes (ISE) to selectively monitor a few chemicals such as chlorine, peroxide, and fluorine.^59,137^ The use of MIP as selective layers in electrodes promises to be the replacement of ISE since this material can be tailor made for each possible application for which it is intended to be applied for.^138,139^ The integration of these advanced sensors would create a system capable of measuring analytes concentration in real time and transfer the data to an IoT cloud server to overcome the limitations of a laboratory based sensing system and offer immediate response for emergencies, processes, and resource management by adopting an evidence based decision making protocol.^64,140^

## 11. Outlook

**Fig. 12:**
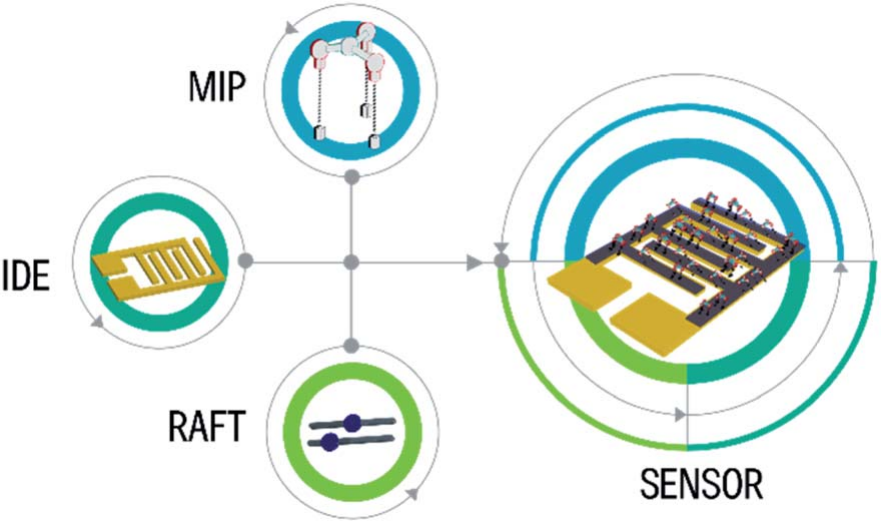
Integration of MIP, RAFT, and IDE for the development of a tailor-made sensor.

According to the present review, a clear trend in the field of molecularly imprinted polymers is identified. Although most previous works reported the use of FRP, more recent works focused on the polymerization design to achieve specific binding events as a tailor made artificial receptor. It is important to highlight that the combination of MIP with RAFT polymerization has taken a route to the synthesis of polymeric brushes by anchoring the CTA in the surface of particles and electrodes to simplify the fabrication process. As mentioned before, the use of electrodes is an attractive manufacturing route because of the large scale production opportunity. This sets a challenge regarding the used materials for these electrodes. This is an opportunity to achieve a market-fit solution regarding sensor fabrication and its implementation in IoT and environmental monitoring applications (Fig. 12).

## Funding

This research received no external funding.

## Author contributions

Conceptualization, I. V., A. S. and J. I.; methodology, J. I., A. S. and I. V.; validation, A. S.; formal analysis, A. S.; investigation, I. V., A. S.; resources, A. S.; data curation, J. I.; writing—original draft preparation, I. V.; writing—review and editing, E. A. and A. S.; visualization, I. V.; supervision, J. I. and A. S.; project administration, A. S.; all authors have read and agreed to the published version of the manuscript.

## Conflicts of interest

The authors declare no conflict of interest. The funders had no role in the design of the study; in the collection, analyses, or interpretation of data; in the writing of the manuscript, or in the decision to publish the results.

## Supplementary Material

RA-012-D2RA00232A-s001
