# Supplementary material for: Molecularly imprinted polymers via reversible addition–fragmentation chain-transfer synthesis in sensing and environmental applications
Source: RSC Adv. 2022 Mar 23;12(15):9186–201. doi: 10.1039/d2ra00232a (PMC8985154; doi:10.1039/d2ra00232a)
Supplement: RA-012-D2RA00232A-s001 [file RA-012-D2RA00232A-s001.pdf]

Electronic Supplementary Material (ESI) for RSC Advances.
This journal is © The Royal Society of Chemistry 2022

Appendix A

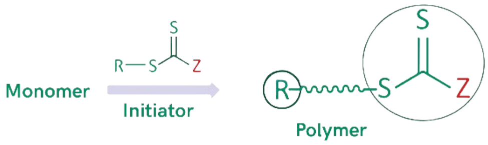

Fig. S1 RAFT initiator control in chain growth over polymerization.

Modified from Sigma Aldrich Copyright, RAFT polymerization products.

Table S1. Summary of Molecularly Imprinted Polymer Reviews in analytical applications.

Application	Scope	Review Study Topic	Experimental Approach	Ref.
Biosensors	Electrochemical biosensors for infectious diseases	MIP as artificial receptors for the detection of infectious diseases	Mechanisms of recognition events between MIP biomarkers.	[53]
	Polymer brushes for drug delivery, biosensing, stimuli responsive and proton conducting	Polymeric structures as selective and responsive to specific stimuli	Implementation of Controlled Radical Polymerization for brushes synthesis	[93]
Environmental monitoring	MIP as sorbents for organic pollutants in wastewater and its treatment	MIP Composites for wastewater treatment	Synthetic route for Core-Shell and CNT composites	[47]
	Experimental conditions for HNT@MIPs microcapsules	Novel material for analytical and environmental monitoring	Hydroponics wastewater treatment	[3]
	MIP-sensor based on: SW/MW-CNT with NPs, Graphene, Graphene/NPs, GO, rGO, CNT/GO/Graphene QDs	MIP and carbon nanomaterials for analytical sensing	Optimization of experimental parameters for sensing	[40]
	Capacitive, CHEMFET, and Fluorescence Quenching Sensing MIPs.	Overview of MIP, from general concepts to Computational modelling	Integration of MIPs as transductors for sensor development	[30]
	Heavy Metal Ions Adsorption Capacity, Detection Limit and of Kinetic Models for Adsorption Isotherms	Principles and techniques of MIPs in the elimination of heavy metal ions	Study of adsorption capacities and reusability for toxicity removal	[94]
	Synthesis in supercritical carbon dioxide ($scCO_2$).	Green approaches to MIP development, from nano- to micron-scale applications.	Improve MIP properties (morphology, homogeneity of binding sites)	[52]
	MIP as dual/multi responsive material in solid-phase extraction, electrodes, and fluorescence sensors	Application of MIP in microextraction, sorption, and biosensors	MIP for in situ analysis of organic and inorganic pollutants	[25]
	N/A	Developments and applications for solid-phase extraction and quantification in complex samples	MIP implementation an extraction material	[79]
	Molecular Imprinting: Green Perspectives and Strategies	Implementation process of green chemistry in MIT: GREENIFICATION	Assistance of upgraded instrumentation in deploying greener goals	[151]
Polymer Science	Specific surface area for MIPs and NIPs	Control over imprinting effect	Determining IP, Pore size distribution of MIP, and NIP	[49]
	Solvent adjustment in MIP-SPE structure, MIP applications ins MIP	MIP synthesis and strategy for pollutants analysis	Optimization of experimental parameters in microextraction	[24]
	N/A	Improving polymerization control to achieve a homogenous network	Design surface modified and uniform MIP nanoparticles	[43]
	Analytical chemistry	Polymers in analytical science with specific molecular architecture and composition	Functional interfaces by CRP approach for analytical applications.	[72]
	Thermodynamic parameters for MIPs.	Intermolecular interactions between the template and the functional monomer.	Analysis of frontier molecular orbitals, electrostatic potentials, and Fukui functions to identify the most stable complex site.	[42]
	N/A	Fine tuning and functionalization of MIP's surface with RAFT fragments.	Protocol to synthesize superparamagnetic MIP using a RAFT polymerization	[95]
	Imprinting methods, templates, and characterization. Comparison of bulk and nanostructured MIP.	Perspectives and applications, of novel preparation technologies and strategies	MIP synthesis strategies for advanced polymer technology	[46]

Table S2. RAFT-MIP works for ion detection review.

Monomer [M]	RAFT Agent [A]	Initiator [I]	[M]:[A]:[I]	Time (hrs)	Material Support	Reference
Methyl methacrylate	CPADB	CdSe QDs	200:01:05	24	QDs	[97]
Methacrylic acid	TTCA	AIBN	55:05:01	8	Fe_3O_4	[109]
Methacrylic acid	DBTTC	AIBN	100:02:01	24	_	[113]
Methyl methacrylate	BDAAT	AIBN	20:03:01	3.5	Graphene Oxide	[98]

Table S3. Monomer and Controlled Radical Polymerization Type reported in MIP analytical applications. Modified from refs. [36,47,56]

Monomer	Abbreviation	CRP employed	Applications	Reference
Cellulose 2,3-bis (3,5- dimethyl phenyl carbamate)-6-acrylate	MON-1	ATRP	Stationary phase for HPLC	[34]
Cellulose 2,3-bis (3,5- dimethyl phenyl carbamate)-6-acrylate	MON-1	ATRP	Stationary phase for HPLC	
1-Vinylimidazole	MON-2	ATRP	Stationary phase for HILIC	
2-hydroxyl-3-[4-(hydroxymethyl)-1H-1,2,3-triazol-1-yl] propyl 2-methylacrylate	HTMA	ATRP	Stationary phase for HILIC	
N-isopropylacrylamide	IPAAm	ATRP	Thermoresponsive stationary phase for HPLC	
3-Sulfopropyl methacrylate potassium	SPM	ATRP	Cation exchange stationary phase for HPLC	
Divinylbenzene	DVB	ATRP	Stationary phase for HPLC	
Butyl methacrylate	BMA	ATRP		
N, N-dimethyl-N-methacryloyloxyethyl-N-(3-sulfopropyl) ammonium betaine	MON-3	ATRP	Membrane for protein binding, stationary phase for HPLC	
2-(2-methoxy ethoxy) ethyl methacrylate	MEO2MA	ATRP	Thermoresponsive monolith for HPLC	
4-Vinylpyridine	4-VP	ATRP	MIP for selective adsorption	
N-tert-butylacrylamide	tBAAm	ATRP	Thermo-responsive stationary phase for HPLC	
Acrylamide	Aam	ATRP	MIP for selective adsorption	[47]
Ethylene glycol methacrylate phosphate	EGMP	ATRP	MIP for selective adsorption	
m-aminophenylboronic acid	APBA-PA	ATRP	Fabricating fluorescent sensor	
Ethylene glycol dimethacrylate	EGDMA	ATRP, RAFT	Stationary phase for HPLC	
Glycidyl methacrylate	GMA	ATRP, RAFT	Restricted access stationary phase for HPLC	
Hydroxyethyl methacrylate	HEMA	ATRP, RAFT	RAM for HPLC	
Methacrylic acid	MAA	RAFT	MIP for selective adsorption	
N, N'-methylenebis (ACRYLAMIDE)	MBA	RAFT	MIP for selective adsorption	[56]
4-((4-Methacryloyloxy)- phenyl azo) benzoic acid	MPABA	RAFT	MIP for selective adsorption	
2-(Dimethylamino)ethyl methacrylate	DMAEMA	RAFT	Thermo- and pH-responsive surfaces	
2,6-bis(acrylamido)pyridine	BAAPy	RAFT	MIP for chiral separation	
N, N-Dimethylacrylamide	DMA	RAFT	Fabricating fluorescent sensor	

Table S4. MIP for ion detection review

Functional monomer	Crosslinking agent	Initiator	Max adsorption capacity	Detected Ion	Limit of detection (sensor)	Reference
Allyl Thiourea	EGDMA	AIBN	5.08 mg/g	As^{+3}	-	[128]
VA	Glutaraldehyde	$NaNO_2$	-	NO^{2-}	10^{-6} M	[107]
MAA	EGDMA	Camphorquinone	26.00 mg/g	Cu^{2+}	-	[101]
Allyl Thiourea	EGDMA	AIBN	11.00 mg/g	NO^{3-}	10^{-5} M	[20]
